# Mapping the Genetic Variation of Regional Brain Volumes as Explained by All Common SNPs from the ADNI Study

**DOI:** 10.1371/journal.pone.0071723

**Published:** 2013-08-28

**Authors:** Christopher Bryant, Kelly S. Giovanello, Joseph G. Ibrahim, Jing Chang, Dinggang Shen, Bradley S. Peterson, Hongtu Zhu, for The Alzheimer's Disease Neuroimaging Initiative

**Affiliations:** 1 Department of Biostatistics, University of North Carolina at Chapel Hill, Chapel Hill, North Carolina, United States of America; 2 Department of Psychology, University of North Carolina at Chapel Hill, Chapel Hill, North Carolina, United States of America; 3 Department of Radiology, University of North Carolina at Chapel Hill, Chapel Hill, North Carolina, United States of America; 4 Biomedical Research Imaging Center, University of North Carolina at Chapel Hill, Chapel Hill, North Carolina, United States of America; 5 The Division of Child and Adolescent Psychiatry, The New York State Psychiatric Institute, New York, New York, United States of America; University G. D'Annunzio, Italy

## Abstract

Typically twin studies are used to investigate the aggregate effects of genetic and environmental influences on brain phenotypic measures. Although some phenotypic measures are highly heritable in twin studies, SNPs (single nucleotide polymorphisms) identified by genome-wide association studies (GWAS) account for only a small fraction of the heritability of these measures. We mapped the genetic variation (the proportion of phenotypic variance explained by variation among SNPs) of volumes of pre-defined regions across the whole brain, as explained by 512,905 SNPs genotyped on 747 adult participants from the Alzheimer's Disease Neuroimaging Initiative (ADNI). We found that 85% of the variance of intracranial volume (ICV) (p = 0.04) was explained by considering all SNPs simultaneously, and after adjusting for ICV, total grey matter (GM) and white matter (WM) volumes had genetic variation estimates near zero (p = 0.5). We found varying estimates of genetic variation across 93 non-overlapping regions, with asymmetry in estimates between the left and right cerebral hemispheres. Several regions reported in previous studies to be related to Alzheimer's disease progression were estimated to have a large proportion of volumetric variance explained by the SNPs.

## Introduction

The heritability of brain structure and function has received extensive attention for many years, and it continues to be an area of active research [1]–[4]. Many studies have used twin designs to estimate the heritability of brain structure and function. Twin studies typically compare the similarity of monozygotic twins (MZ), who share the same genetic materials, to that of dizygotic twins (DZ), who share 50% of their genes. Assuming equal environmental exposure across zygosities, the known differences in genetic similarity allow us to distinguish the effects of genes and environment on a phenotype, including a phenotype characterized using brain imaging measures.

Genome-wide association studies (GWAS) have discovered hundreds of single-nucleotide polymorphisms (SNPs) that are significantly associated with variation in brain structure and function [5]–[11]. A study using the ADNI database found two SNPs associated with temporal lobe volume [12] while two other ADNI studies found no SNPs associated with voxelwise volume differences, after multiple comparison adjustment [13], and no significant genes in a multivariate analysis combining the SNPs into genes [14]; five SNPs were found to be significant in the ADNI population for 142 various imaging phenotypes [15]. In cases where significant SNPs are found in GWAS, however, those SNPs typically explain only a small portion of overall phenotypic variation. A question that has not been addressed fully for phenotypic brain measures is where in the genome the missing heritability resides. Explanations could include that causal variants each may explain such a small amount of variation that their effects do not reach stringent significance thresholds and/or that the causal variants may not be in complete linkage disequilibrium (LD) with the SNPs that have been genotyped. Heritability is defined as the proportion of observed phenotypic variation that is due to inherited genetic factors; GCTA (genome-wide complex trait analysis) software allows us to approximate heritability with the proportion of phenotypic variance explained by variation among over 500,000 SNPs, after adjusting for environmental factors. GCTA has been utilized previously to partition the genetic variation of complex traits such as height and BMI into contributions from each chromosome [16] and to estimate the missing heritability of disease from GWAS [17].

Here we estimate the proportion of variance of 93 regional volumes, as well as ICV, GM, WM, and cerebro-spinal fluid (CSF) volumes, explained by 512,905 SNPs across the 22 autosomes genotyped on 747 adult participants from the ADNI study.

## Materials and Methods

### Sample and Data

The NIH ADNI is an ongoing public-private partnership to test whether genetic data, structural and functional neuroimaging, and clinical data can be combined to measure the progression of mild cognitive impairment (MCI) and early Alzheimer's disease (AD). The structural brain MRI data and corresponding clinical and genetic data from baseline and follow-up were downloaded from the ADNI publicly available database (http://adni.loni.ucla.edu/). This initiative is a collective effort led by Principal Investigator Michael Weiner, M.D., VA Medical Center and University of California – San Francisco, involving many co-investigators and recruitment of participants from over 50 sites in the United States and Canada. More information on the study can be found at www.adni-info.org.

The Human 610-Quad BeadChip (Illumina, Inc., San Diego, CA) was used to genotype 818 participants in the ADNI database, which resulted in a set of 620,901 SNP and copy number variation (CNV) markers. Since the Apolipoprotein E (APOE) SNPs, rs429358 and rs7412, are not on the Human 610-Quad Bead-Chip, they were genotyped separately. These two SNPs together define a 3 allele haplotype, namely the e2, e3, and e4 variants, and the presence of each of these variants was available in the ADNI database for all the individuals; the APOE gene has been the most significant risk locus in GWAS of AD [18]. The software EIGENSTRAT in the package of EIGENSOFT 3.0 was used to calculate the population stratification coefficients of all participants. To reduce population stratification effects, we initially used 747 Caucasians out of the total 818 participants.

The MRI data were collected across a variety of 1.5 Tesla MRI scanners with protocols individualized for each scanner, including volumetric 3-dimensional sagittal MPRAGE or equivalent protocols with varying resolutions. The typical protocol included: repetition time (TR) = 2400 ms, inversion time (TI) = 1000 ms, flip angle = 8°, field of view (FOV) = 24 cm, with a 256*256*170 acquisition matrix in the x-,y-, and z-dimensions yielding a voxel size of 1.25*1.26*1.2 mm^3^. All original uncorrected image files are available to the general scientific community, as described at http://www.loni.ucla.edu/ADNI.

### Image preprocessing and analysis

The MRI data were preprocessed using standard procedures that included realignment to the anterior commissure and posterior commissure by using MIPAV software, skull-stripping by using Brain Surface Extractor (BSE) and Brain Extraction Tool (BET), cerebellum removal, intensity inhomogeneity correction, segmentation using the FSL-FAST software, and spatial co-registration by using HAMMER [19]–[21]. Particularly, to establish the longitudinal correspondences in the individual and the inter-subject correspondences between the template and the individual, we combined information across time points for each subject and registered the images to a Jacob template [22] using a fully automatic 4-dimensional atlas warping method called 4D HAMMER [21]. Regional volumetric measurements and analyses are then performed via measurements and analyses of the resulting tissue density maps. Lastly, we carried out automatic regional labeling: first, by labeling the template image and second, by transferring the labels following the deformable registration of subject images. After labeling 93 regions from the Jacob atlas, we were able to compute volumes for each of these regions for each subject.

### Statistical Methods

GCTA (genome-wide complex trait analysis) [23] software running a linear mixed model (LMM) was used to estimate the proportion of variance of an observed phenotype that was explained by a set of SNPs (rather than a single SNP, as in GWAS), which we call genetic variation for simplicity. Here the phenotypes of interest are the 93 regional volumes as well as total intracranial (ICV), grey matter (GM), white matter (WM), and cerebrospinal fluid (CSF) volumes, and the SNPs numbered 512,905 from 747 participants. Genetic relationship matrices (GRMs) captured correlations across participants and were then used to partition the phenotypic variance (see [23] for details). Via GCTA, we fitted LMM separately to each of the 97 volumes as dependent variables to estimate their genetic variation, as explained by the 512,905 SNPs through GRMs. The volumes were standardized to better fit the normal distribution assumed for the LMM, and baseline age, gender, and the interaction of age with gender were included as covariates, to capture variation due to environmental effects. To adjust for population structure, we also included subjects' first ten principal components of the GRM as covariates. ICV was also included as a covariate (for regions other than ICV itself) to remove any potential scaling effects. Within GCTA, we corrected for imperfect LD between the SNPs on the array and causal variants by assuming the same allelic distribution (i.e. minor allele frequency) for those causal variants (which were potentially not in the SNP array) as that observed in the included SNPs. See [24] for details on how the correction is implemented within the GCTA software. This correction allows for better approximation to true heritability within this observational study.

An R package, called APCluster, was used to group standardized volumes into groups of similar regions based on the GCTA measures. Affinity propagation (AP) clustering [25]–[26] was applied first to the 93 non-overlapping regional (scaled and standardized) volumes separately for each diagnostic group. Clustering was based on the negative squared Euclidean similarity matrix between the regional volumes, finding “exemplars” that were the centers of the clusters and iteratively determining the number and membership of the clusters to maximize their “net similarity,” an objective function that measures how representative the exemplars are of the data in each cluster. This technique was then applied to cluster the genetic variation estimates for the 93 non-overlapping regions. The vector of the proportions of volumetric variance explained by all SNPs (after adjusting for covariates) for the 93 non-overlapping regions was clustered based on the squared distances of these genetic variation estimates. Fisher's exact tests were used to compare clustering results: pair-wise between study groups and volumetric clustering compared to the clustering of genetic variation estimates.

## Results

**Figure 1:**
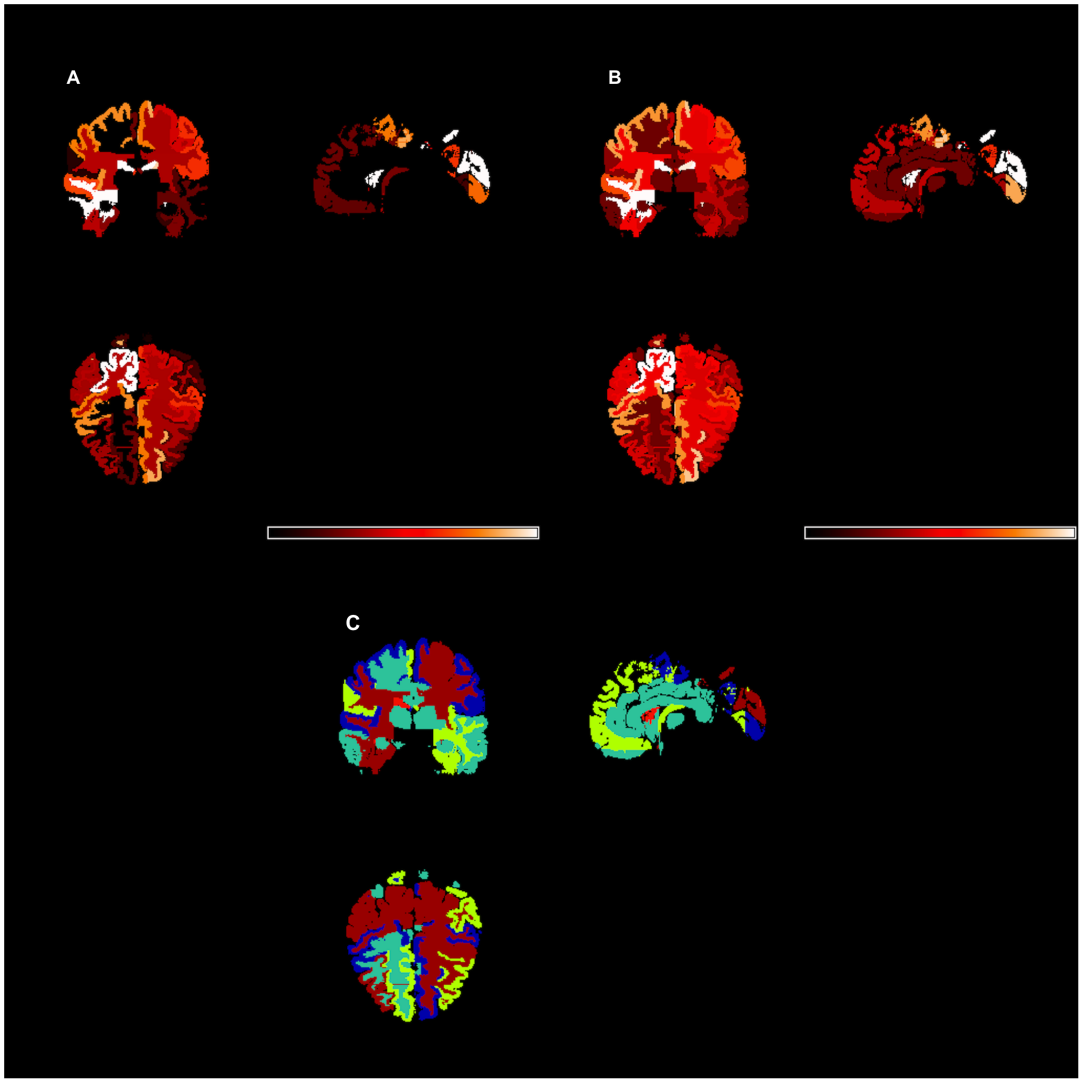
Genetic variation estimates and additional results for non-overlapping brain regions. Genetic variation estimates (top left; A) and the associated −log_10_p-values from LRT (top right; B). Hotter colors (black<red<white) indicate larger values. Clusters of genetic variation estimates (below center; C) using AP cluster method.

**Figure 2:**
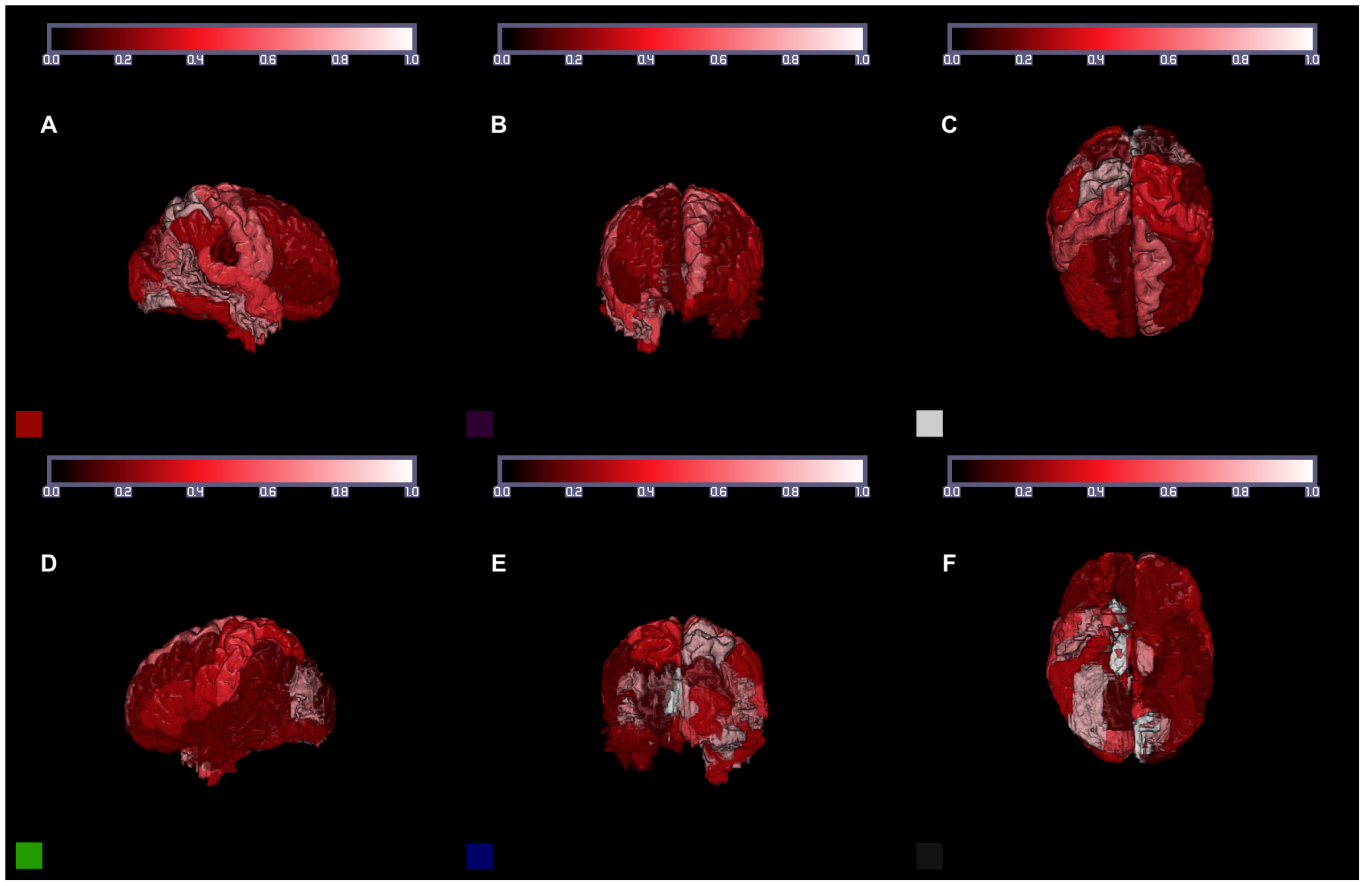
3-dimensional map of genetic variation estimates. Views (clockwise from top left): right lateral (A), anterior (B), superior (C), inferior (F), posterior (E), and left lateral (D). Hotter colors (black<red<white) indicate larger values.

Table 1 gives demographic information on the participants used in these analyses. Age is similarly distributed for each of the three study groups and there is a significant association between gender and study group (p = 0.02); both age and gender were adjusted for in each analysis. Tables 2 and 3 present genetic variation estimates, their standard errors, the p-values from the associated likelihood ratio tests (LRTs), and clustering results of the estimates for all regions. Figure 1 shows genetic variation estimates (top left) and the corresponding −log_10_p-values from the likelihood ratio tests (top right) for the standardized volumes of each non-overlapping region as explained by the 512,905 SNPs across the entire genome, after adjusting for covariates. Figure 2 shows genetic variation estimates in three dimensions. Genetic variation estimates were found to be uncorrelated with average region size (p = 0.2; from GLM with logit link function, Normal distribution).

**Table 1:** Participants' gender and baseline age by study group.

	*Gender*			*Age*					
	Male	Female	Total	Min	1st quartile	Median	Mean	3rd quartile	Max
**Normal**	111	95	206	60	73	76	76	79	90
**MCI**	233	129	362	55	71	75	75	80	90
**Alzheimer's**	98	81	179	55	71	77	75.5	81	91
**Total**	442	305	747	55	71	76	75.4	80	91

Gender and baseline age distribution by study group. Chi-squared test of independence between gender and study group yields a p-value of 0.02. ANOVA F-test for differences in mean age between study groups yields a p-value of 0.18.

**Table 2:** Genetic Variation estimates for major regional brain volumes.

Region	Estimated genetic variation	Standard error	LRT p-value
ICV	0.845	0.457	0.04
GM Volume	<0.001	0.476	0.5
WM Volume	<0.001	0.483	0.5
CSF Volume	0.574	0.468	0.12

Genetic variation estimates, standard errors, and associated likelihood ratio tests for four aggregated volumes.

**Table 3:** Genetic variation estimates and associated clustering results for ROI volumes.

Region	Genetic Variation	SE	LRT p-value	Cluster
lateral ventricle right	0.999	0.468	0.017	1
globus palladus right	0.907	0.440	0.024	1
caudate nucleus right	0.999	0.459	0.006	1
cuneus left	0.999	0.462	0.010	1
nucleus accumbens left	0.894	0.494	0.053	1
lateral ventricle left	0.729	0.476	0.080	2
caudate nucleus left	0.679	0.458	0.073	2
temporal lobe WM right	0.743	0.473	0.068	2
occipital lobe WM left	0.754	0.450	0.055	2
superior parietal lobule right	0.822	0.442	0.038	2
lateral occipitotemporal gyrus right	0.833	0.472	0.046	2
entorhinal cortex right	0.785	0.442	0.040	2
cuneus right	0.766	0.455	0.050	2
insula right	0.595	0.449	0.094	3
precentral gyrus right	0.589	0.478	0.117	3
medial frontal gyrus left	0.563	0.468	0.120	3
globus palladus left	0.581	0.483	0.127	3
putamen right	0.590	0.470	0.108	3
subthalamic nucleus right	0.586	0.465	0.105	3
occipital lobe WM right	0.628	0.473	0.102	3
precuneus left	0.477	0.498	0.191	3
superior frontal gyrus left	0.626	0.462	0.094	3
postcentral gyrus left	0.467	0.476	0.169	3
perirhinal cortex right	0.544	0.464	0.122	3
postcentral gyrus right	0.569	0.459	0.110	3
lingual gyrus right	0.542	0.446	0.108	3
superior temporal gyrus right	0.499	0.486	0.165	3
fornix right	0.472	0.451	0.142	3
middle frontal gyrus right	0.263	0.491	0.306	4
inferior frontal gyrus left	0.266	0.456	0.276	4
angular gyrus right	0.269	0.483	0.296	4
frontal lobe WM left	0.274	0.475	0.286	4
posterior limb of internal capsule inc. cerebral peduncle left	0.334	0.492	0.261	4
posterior limb of internal capsule inc. cerebral peduncle right	0.411	0.486	0.212	4
superior parietal lobule left	0.320	0.478	0.260	4
parietal lobe WM left	0.276	0.499	0.303	4
precentral gyrus left	0.327	0.490	0.263	4
medial front-orbital gyrus left	0.392	0.456	0.195	4
parietal lobe WM right	0.291	0.474	0.273	4
parahippocampal gyrus right	0.230	0.466	0.310	4
occipital pole right	0.288	0.472	0.275	4
inferior temporal gyrus right	0.293	0.474	0.270	4
medial front-orbital gyrus right	0.176	0.468	0.353	5
superior frontal gyrus right	0.153	0.497	0.386	5
putamen left	0.182	0.465	0.346	5
parahippocampal gyrus left	0.112	0.483	0.411	5
fornix left	0.205	0.482	0.341	5
precuneus right	0.148	0.489	0.386	5
superior occipital gyrus right	0.135	0.453	0.379	5
supramarginal gyrus left	0.157	0.482	0.376	5
middle frontal gyrus left	0.160	0.481	0.373	5
supramarginal gyrus right	0.085	0.489	0.434	5
inferior frontal gyrus right	0.179	0.476	0.356	5
temporal lobe WM left	0.183	0.475	0.352	5
lateral front-orbital gyrus left	0.188	0.474	0.348	5
insula left	0.156	0.458	0.365	5
medial frontal gyrus right	0.190	0.467	0.343	5
angular gyrus left	0.122	0.462	0.395	5
medial occipitotemporal gyrus right	0.131	0.473	0.392	5
lateral occipitotemporal gyrus left	0.217	0.470	0.323	5
occipital pole left	0.104	0.486	0.419	5
lateral front-orbital gyrus right	<0.001	0.458	0.500	6
cingulate region right	<0.001	0.478	0.500	6
frontal lobe WM right	<0.001	0.471	0.500	6
temporal pole right	<0.001	0.484	0.500	6
nucleus accumbens right	0.059	0.488	0.454	6
uncus right	<0.001	0.490	0.500	6
cingulate region left	<0.001	0.472	0.500	6
subthalamic nucleus left	<0.001	0.466	0.500	6
hippocampal formation right	<0.001	0.498	0.500	6
inferior occipital gyrus left	<0.001	0.457	0.500	6
anterior limb of internal capsule left	<0.001	0.486	0.500	6
superior temporal gyrus left	<0.001	0.472	0.500	6
uncus left	<0.001	0.473	0.500	6
middle occipital gyrus right	<0.001	0.471	0.500	6
middle temporal gyrus left	<0.001	0.476	0.500	6
lingual gyrus left	<0.001	0.425	0.500	6
perirhinal cortex left	<0.001	0.478	0.500	6
inferior temporal gyrus left	<0.001	0.495	0.500	6
temporal pole left	<0.001	0.479	0.500	6
entorhinal cortex left	<0.001	0.491	0.500	6
inferior occipital gyrus right	<0.001	0.463	0.500	6
superior occipital gyrus left	0.060	0.473	0.450	6
hippocampal formation left	<0.001	0.479	0.500	6
thalamus left	<0.001	0.474	0.500	6
amygdala left	<0.001	0.484	0.500	6
medial occipitotemporal gyrus left	<0.001	0.478	0.500	6
anterior limb of internal capsule right	<0.001	0.486	0.500	6
middle temporal gyrus right	<0.001	0.480	0.500	6
corpus callosum	<0.001	0.488	0.500	6
amygdala right	<0.001	0.505	0.500	6
middle occipital gyrus left	<0.001	0.460	0.500	6
thalamus right	<0.001	0.473	0.500	6

Genetic variation estimates, standard errors, associated LRT p-values, and clustering results for 93 non-overlapping ROIs.

The AP cluster method identified 9, 10, and 10 clustering groups within the 93 non-overlapping regional volumes for the Control, MCI, and AD groups, respectively. Fisher's exact tests of independence yielded p<0.001 for each of the pairwise comparisons of clustering membership, indicating similarity between the volumetric clustering results across the three groups. Subsequently all participants were combined into a single analysis, yielding 9 clusters of regional volumes. In addition, AP clustering identified 6 groups of (non-overlapping) regions based on genetic contributions to variation in their standardized, covariate-adjusted volumes (see Table 2). Figure 1 shows the clustering map (bottom) for all of the non-overlapping regions. A Fisher's exact test yielded a p-value of 0.22 when testing for independence between the two sets of clustering results (from the raw volumes and from the genetic variation estimates), leading us to conclude that the groups that are similar in volume are not necessarily similar in the proportion of volumetric variance explained by the SNPs.

## Discussion

GCTA findings showed that about 85% of intra-cranial volume (ICV) variability and 57% of cerebrospinal fluid (CSF) volume variability were explained by genetic variation within these participants, with likelihood ratio tests (LRT) for the significance of the random component yielding p-values of 0.04 and 0.12, respectively. However, the genetic variation estimates for total grey matter (GM) and white matter (WM) volumes (after adjusting for ICV and other covariates) were both near zero. Previous studies of adults reported similar estimates for ICV heritability (73% in [27]; 81% in [28]; 79% in [29]; See [30] for a review). Carmelli et al. [27] estimated heritability of CSF volumes to be 72% in their study of normal elderly twins, similar to our estimates. We found markedly less genetic variation in our sample for GM and WM volumes than did previous studies (WM: 73% heritable in [27]; 62% in [29]; 69%–82% in WM regions and 55–85% in GM regions in [4]), likely because of our adjusting for ICV to remove scaling effects. Analysis of GM and WM volumes, without adjusting for ICV, yielded genetic variation estimates of 41% (LRT p-value = 0.21) and 91% (LRT p-value = 0.02) respectively, which are more consistent with published heritability estimates, indicating that some of the previously published heritability estimates for these volumes may be due to the heritability of overall brain volume rather than genetic contributions that are specific to GM or WM.

In regional analyses, we found estimates that were asymmetric across hemispheres, with no clear systematic difference between left hemisphere and right hemisphere volumes. This is in agreement with one prior report [31] and in a twin study of schizophrenia [4] but at odds with another study that reported symmetric heritability in only 10 monozygotic and 10 dizygotic twin pairs, a sample that likely afforded insufficient statistical power to detect differences in heritability across hemispheres [32]. Chen et al. [1] found bilateral symmetry across hemispheres in their mapping of the brain's cortical surface, but their study involved 406 healthy twins and did not consider regional volumes. We found the left and right cuneus to have significant genetic variation (estimates of 99%, p = 0.01 and 77%, p = 0.05, respectively); Niskanen et al. [33] found the cuneus to be involved in the progression of AD, which could explain the genetic variation in our study population. We also found the right caudate nucleus to have significant genetic variation (estimate of 99%, p = 0.02; left caudate nucleus: estimate of 68%, p = 0.07); this is a region found previously to be significantly smaller in AD patients than in normal controls [34], including in the ADNI population [35], suggesting that size differences in the right hemisphere in AD may be heritable.

The AP cluster analyses indicated the presence of 6 groups of brain regions within the genetic variation estimates; the largest group was comprised of those regions with little to no volumetric variability explained by the SNPs. The clusters of regional volumes differed significantly from the clusters of the corresponding genetic variation estimates, but average regional volumes were not associated significantly with genetic variation estimates. Thus, the regions that were similar in size were not necessarily similar in genetic variation, and there was no apparent relationship between a region's size and the genetic variation of the region.

In the current study, we systematically estimated the variability of 93 non-overlapping regional volumes, ICV, and GM, WM, and CSF volumes explained by 512,905 SNPs across the 22 autosomes genotyped on 747 adult participants from ADNI. These results give some evidence for a genetic component of the variation between individuals in several functional brain systems.

First, there were several medial/mid-line brain structures with high genetic variation estimates, including the bilateral medial frontal-orbital gyrus, left medial frontal gyrus, bilateral precuneus, right perirhinal cortex and right entorhinal cortex. These structures, along with other medial neural regions, have been characterized as being part of the default mode network (DMN) - defined as a set of functionally connected brain regions that exhibit task-induced deactivation and increase activity at rest [36]–[37]. DMN changes have been observed in MCI and AD, particularly in the posterior region of medial parietal cortex referred to as the precuneus [38]. Additionally, studies of resting glucose metabolism have demonstrated hypometabolism in the inferior parietal lobule that progresses with AD and correlates with mental status [39]–[40] and is present in individuals at genetic risk for AD [41].

Second, many subcortical nuclei, as well as white matter tracts, had large genetic variation estimates, including the right insula, bilateral globus pallidus, bilateral putamen, bilateral caudate and left nucleus accumbens. Many of these structures make up the basal ganglia (i.e., globus pallidus, putamen, caudate nucleus, nucleus accumbens), a group of nuclei that act as a cohesive functional unit. The basal ganglia are associated with a variety of functions, including motor control, procedural learning relating to routine behaviors or “habits”, eye movements, and emotional functions [42]. Additionally, several theories implicate the basal ganglia in action selection (i.e., deciding which of several possible behaviors to execute at a given time [43]–[44]). Beyond the basal ganglia, another subcortical region with high estimated genetic variation was the insula, a structure believed to play a role in functions usually linked to emotion (i.e., disgust) or the regulation of the body's homeostasis. Although changes in subcortical regions are not the hallmark of MCI or AD, as AD is considered a cortical dementia, the large estimates observed in the current study indicate that future research in this area is warranted.

Finally, large genetic variation estimates were observed for two prominent perceptual cortical pathways, namely the dorsal “where” pathway and the ventral “what” pathway. First described by Ungerlieder and Mishkin [45], the dorsal pathway leads from striate cortex to the parietal lobe and is responsible for determining an object's location in space, whereas the ventral pathway leads from striate cortex to the temporal lobe and is responsible for determining an object's identity. Via an interaction between bottom-up (visual perceptual information) and top-down (knowledge information) processing these pathways are utilized to rapidly to perceive “what” objects are in an environment and “where” those objects are spatially located relative to the observer. Given the clear adaptive nature of such visual processing streams, it is perhaps unsurprising that large genetic variation estimates were observed for several key regions that comprise these pathways, including bilateral occipital white matter tracts, bilateral occipital pole, bilateral temporal lobe white matter tracts, bilateral parietal lobe white matter tracts, bilateral superior parietal lobule and bilateral occipito-temporal gyrus.

Due to a lack of power in the likelihood-ratio testing procedure and a sample size that was not sufficiently large to make standard errors small, we did not find estimates to be highly statistically significant. We did not perform multiple comparisons adjustments on the p-values from these analyses, in order to preserve the ability to compare results across different brain regions. Here our goal is to map genetic variation across the entire brain, where the results of particular interest are the comparisons of genetic variation among different regional volumes. The mixed model approach utilized by GCTA allows for an approximation to total heritability based on a large number of SNPs; this methodology is validated by the comparison of our results to published twin studies that estimate “true” heritability and have similar findings. Estimates of 0.999 for three regions do not imply that over 99% of variation in those volumes is explained by genetic variability, but they likely indicate large true parameter values (i.e. volumetric variance explained by the SNPs) and some additional variation due to the (case-control) sampling scheme, as well as noise in the volumetric data.

Although our findings agree with those from a number of published twin studies, some of our findings could derive from our study population, in that some of the genetic contribution to the progression of AD could have contributed to the genetic variation we detected. We found significant but asymmetric genetic variation estimates in regions previously reported to be related to AD, and we found that much of the heritability of overall GM and WM volumes may be due to the heritability of total ICV rather than from specific genetic contributions to GM or WM volumes. In the future, large twin studies in elderly patients could compare these regions in their heritability and assess their relationship with the progression of Alzheimer's disease.
